# Supplementary material for: Analysis of PPARγ Signaling Activity in Psoriasis
Source: Int J Mol Sci. 2021 Aug 10;22(16):8603. doi: 10.3390/ijms22168603 (PMC8395241; doi:10.3390/ijms22168603)
Supplement: Supplementary file 1 [file ijms-22-08603-s001.zip › Supplemental materials_Analysis of PPARg signaling activity in psoriasis/Pathway models/Models images and html files/Differentiation of psoriatic T cells/189826.html]

Th1 immune response


# Cell Process Th1 immune response

|  |  |
| --- | --- |
| URN | urn:agi-gocellproc:0042088 |
| Total Entities | 0 |
| Connectivity | 441 |
| Name | Th1 immune response |

---

|  |  |
| --- | --- |
| Pathway | DCIR1 (CLEC4A) Signaling |
|  | Macrophage M1 Lineage |
|  | AHR Signaling in M1 Macrophages Function |
|  | Dendritic Cells Function in Atherosclerosis |
|  | T-Cells Differentiation Block in Psoriasis |
|  | Dendritic Cells Function in Psoriasis |
|  | Th0-Cell Aberrant Activation in Systemic Lupus Erythematosus |
|  | Dendritic Cell Dysfunction in Crohn's disease |
|  | Th2-Cell Function in Ulcerative Colitis |
|  | Th17-Cell and Th1 Immune Responsein Psoriatic Arthritis |
|  | IL6/IL12 Signaling Activates Immune System in Multiple Sclerosis |
|  | PPAR Psoriasis |
|  | New Pathway (3) |
|  | 1\_Differentiation of psoriatic T cells |
|  | 1\_Defects in response to pathogens in the gut promotes inflammation in Crohn's disease |
|  | 2\_Intestine epithelial cells drive immunological responses in ulcerative colitis |
|  | Differentiation of psoriatic T cells |

---

|  |  |
| --- | --- |
| MedScan ID | 4142088 |

---

|  |  |
| --- | --- |
| Alias | Th1 immune response |

---

|  |  |
| --- | --- |
| GO ID | 0042088 |

---
